# Supplementary material for: Patterns of Intron Gain and Loss in Fungi
Source: PLoS Biol. 2004 Nov 30;2(12):e422. doi: 10.1371/journal.pbio.0020422 (PMC532390; doi:10.1371/journal.pbio.0020422)
Supplement: Table S1 — Also available at http://genes.mit.edu/NielsenEtAl/. (4.3 MB ZIP). [file pbio.0020422.st001.zip › NielsenEtAl/html/1191.html]

AN4268.1.NCU03949.1.MG07261.1.FG10065.1


```
 CLUSTAL W (1.82) Multiple Sequence Alignments - Introns Inserted


Sequence 1: NCU03949.1	378 aa
Sequence 2: MG07261.1	325 aa
Sequence 3: FG10065.1	354 aa
Sequence 4: AN4268.1	342 aa
Alignment Length: 387 aa
Number Identitical Residues: 76 aa
Alignment Score (without introns) 4576


MG07261.1 	--------------MATTTLLKKWFPHTEAPVIISAPMLGIATGTLAAQVTKAGGLG1MV
NCU03949.1	MHFPGHSSKKEESAQAALTKLNSWFPTTKNPVIISAPMYLIANGTLAAEVSKAGGIG1FV
FG10065.1 	---------------MSPSQLLSRFPWATSPLISNAPMLGIATPRMAAEVTKAGGLG1FL
AN4268.1  	--------------MSTTQALQTSYPWTKMPLLASAPMLNIASAQLAVSVSAAGGLG~FL
          	                :   * . :* :  *:: .***  **.  :*..*: ***:* ::

MG07261.1 	PGGYDVTPGSTQLAELDAELSTVRQVLGLQTS-SDPLP-VGIGFICCHGIVDKHFGGRGL
NCU03949.1	AGGSDFRPGSSHLTALSTELASARSRLGLTDRPLTPLPGIGVGLILTHTISVPYVTDTVL
FG10065.1 	PCVAYIEPNSEHVNRLADHFDEVRALLGAGHSSDGTLP-VGVSFITSHSSMS-YFEETAL
AN4268.1  	AGGFDVSSLESNLEEAVQLFKQSNSPAQQNYAASGMLP-IGIGFLNWGADLPRSIAA---
          	.    . . . ::      :   .        .   ** :*:.::         .     

MG07261.1 	ADLVAKHRPAAVWLFAPEP--DAGYEKLVR--AARDAAAAAGYEVRVFAQ~VGTVADARR
NCU03949.1	P-ILIEHSPQAVWLFANDPDFEASSEPGAKGTAKQIIEALHASGFVVFFQ~VGTVKDARK
FG10065.1 	P-LVVKNRPAAVWLFAPDGDLKPHSKIIPS-------LKRLEYPPIVFVQ~VGNVAVARE
AN4268.1  	---IANYRPCAVWFFGAKAIPEDLRP--------------------WFEQ0VGTVSEAVA
          	   : :  * ***:*. .   .                         * * **.*  *  

MG07261.1 	AVAAG-VDVVVAQGVDAGGHQFAVGSGIVGLVPEVRDMVDDEFPGKEVAVVAAGGIADGR
NCU03949.1	AAADG-ADVIVAQGIDAGGHQLATGSGIVSLVPEVRDMLDREFKEREVVVVAAGGVADGR
FG10065.1 	AVEDG-ADVLVTQGVDAGGHQFRQGSGIVSLVPEVRDMLSREFPDRGISIVAAGGIADGR
AN4268.1  	VAESLSPDALVVQGSDAGGHGLTNSASIITLVPEVKDALEARQLRDHIPIIAAGGIVDGR
          	..   : *.:*.** ***** :  .:.*: *****:* :. .     : ::****:.***

MG07261.1 	GVAAALALG1AEGAVMGTR0FLVADEAKTADWQRKMVLETRDGGLNTNK2SVFHDQLRGT
NCU03949.1	GVVGALGLG1AEGVVLGTR0FTVAVEASTPEFRRKVILETNDGGLNTVK2SHFHDQIN-C
FG10065.1 	GVAGALALG1AEGVVMGTK0FTIAPESNYPEIRKQKVLEAVDGGVSTFK2STFNDRIT-N
AN4268.1  	GLAASLVLG~ASGAAMGTR~FLASSEARIARGYQDEVLRASDGGVSTVR~STVYDRVR-G
          	*:..:* ** *.*..:**: *  : *:  .   :. :*.: ***:.* : * . *::   

MG07261.1 	DSLWPPFYDGRAIVTDSYRDHRV-GVSLEENKKRFNQAKESGDNSS----RGVVWS~---
NCU03949.1	NTIWHNVYDGRAVRNASYDDHAA-GVPFEENHKKFKEAASSGDNS-----RAVTWS2GTA
FG10065.1 	SPLWGPLYDGRAIVGPIHEKFIA-GASLEECQRSLKEDYHEDEARY----IINTWA2GTG
AN4268.1  	ILSWPSRYDGRGVINQTYIDAVERGMSDEENKALYEEELKKGDSGWGPNARLTTYA~GTG
          	   *   ****.:    : .    * . ** :   ::   ..:   ...:   .:: .:.

MG07261.1 	---------------------------------
NCU03949.1	VGLIKDQRPAGDIVRELREEAKERIKKIQAFAA
FG10065.1 	VGLINKVQPAGEIVREVREETKREIQRAAGLV-
AN4268.1  	LGLVREILPAADIVANVIREAESTLQGLKV---
          	 .   .  .:..   .   .:.   .
```
